# Radiographic assessment of radiolucent lines around a highly porous titanium cup (Tritanium) using digital tomosynthesis, after total hip arthroplasty

**DOI:** 10.1186/s13018-021-02396-4

**Published:** 2021-04-15

**Authors:** Kazuki Oishi, Yuji Yamamoto, Yoshifumi Harada, Ryo Inoue, Eiji Sasaki, Yasuyuki Ishibashi

**Affiliations:** Department of Orthopaedic Surgery, Hirosaki University Graduate School of Medicine, 5 Zaifu-cho, Hirosaki, Aomori, 036-8562 Japan

**Keywords:** Highly porous titanium cup, Digital tomosynthesis, Hip arthroplasty, Radiolucent lines

## Abstract

**Background:**

The objectives of this study were to assess radiolucent lines around a highly porous titanium cup (Tritanium) using digital tomosynthesis and to investigate the clinical and radiographic factors associated with radiolucent lines on tomosynthesis.

**Methods:**

Fifty-five patients underwent total hip arthroplasty using a Tritanium cup, and digital tomosynthesis and plain radiography were performed at 1 week, 6 months, 1 year, and 2 years after surgery. The radiolucent lines around the cup were measured on both DTS and plain radiography at each postoperative period. Clinical evaluations were performed by the Japanese Orthopaedic Association hip disease evaluation questionnaire (JHEQ), and revision surgeries were examined. Based on the presence of radiolucent lines on digital tomosynthesis at 2 years postoperatively, patients were divided into RL (+) and RL (−) groups and investigated for related factors.

**Results:**

There were 20 cases in the RL (+) group and 35 cases in the RL (−) group, and no revision surgeries were required. Statistically, there were more cases with radiolucent lines on digital tomosynthesis (45.4% at 1 week and 36.3% at 2 years) than on plain radiography (9.1% at 1 week and 9.1% at 2 years) at each postoperative point. Logistic analysis showed no significant associations between the presence of radiolucent lines at 2 years on digital tomosynthesis, and the JHEQ parameters of pain (*p* = 0.937), movement (*p* = 0.266), or mental status (*p* = 0.404).

**Conclusion:**

In a short-term evaluation up to 2 years, digital tomosynthesis detected more radiolucent lines around the titanium cups than plain radiography. The occurrence of radiolucent lines was not related to the postoperative clinical evaluation.

## Background

Implant failures in primary total hip arthroplasty (THA) requiring revision surgeries continue to occur, mostly as a result of aseptic loosening, infection, or instability. In the USA, there were 50,220 revision THAs in 2014, and the incidence is projected to increase by somewhere between 43 and 70% from 2014 to 2030 [1]. Aseptic loosening of the acetabular implant is one of the leading causes of early implant failure in primary THA, necessitating a revision surgery [2]. Highly porous cups for THA were developed in order to improve the osseointegration, enhance the long-term durability, and reduce the rates of aseptic loosening and delamination to a minimum [3]. Excellent short- to medium-term clinical results have been reported in primary THA using a highly porous cup, with a 0.7% revision rate and a 0.1% rate of aseptic loosening [4].

The Tritanium primary cup (Stryker, Mahwah, NJ) is a highly porous 3-dimensional (3D) structure designed to mimic the trabecular morphology of natural bone. The Tritanium cups exhibited a 23–65% improvement in initial stability when compared to conventional plasma-sprayed cup designs [5] and sought to improve osseointegration, reduce stress shielding, and increase stability [6]. The postoperative radiographic evaluation of highly porous cups has been reported in various papers. Some studies showed no cup migration or any signs of radiolucent lines (RLs) [3, 7], and others reported high rates of RLs at both the short- and medium-term [6, 8]. Yoshioka et al. reported that RLs were found in 36.1% of the primary Tritanium-treated patients at 3 months, increasing to 60% at 41 months, which was the final follow-up [8].

In previous studies, postoperative imaging evaluation for highly porous cups has been performed by plain radiography and computed tomography (CT). There are several problems with these, including difficulties in making a detailed assessment on plain radiography, and metal artifacts on CT imaging. Digital tomosynthesis (DTS) is a new technology that has been introduced for clinical practices such as breast cancer diagnosis [9], and there have also been several studies on its use after hip and knee arthroplasty [10, 11]. In cementless THA, the sensitivity of DTS to detect osteointegration has been reported to be superior to that of plain radiographs and conventional CT [10]. However, there are few studies that have used DTS to detect RLs in the early postoperative evaluation of THA using the Tritanium cup.

The objectives of this study were to assess RLs around a highly porous titanium cup (Tritanium) using DTS and to investigate the clinical and radiographic factors associated with RLs. We hypothesized that DTS could identify RLs around a highly porous titanium cup better than plain radiography.

## Methods

### Patient population

From June 2015 to March 2018, 75 consecutive patients (80 hips) diagnosed with hip osteoarthritis (OA) and osteonecrosis (ON) of the femoral head were treated with primary THA. Nine patients who had already received THA on the other side, 5 patients (10 hips) who received bilateral THAs within the same period, 4 patients who could not be followed for 2 years after surgery, and 2 patients who had previously undergone an acetabular rotational osteotomy were excluded from this study. The remaining 55 patients (11 males and 44 females; 55 hips) were surgically treated using a highly porous titanium cup (Tritanium, Stryker) and were followed up for at least 2 years. The demographic data of all 55 patients were collected, including their age at the time of surgery, sex, body mass index (BMI), preoperative diagnosis, and surgical approach.

This study was approved by the institutional review board of our institution (2020-081). All participants provided written informed consent. This study was conducted in accordance with the principles outlined in the Declaration of Helsinki (1964) and revised in 2013.

### Surgical procedure

Regarding the operative approach for THA, an anterolateral approach in the supine position (AL-S) was employed for 43 patients, and a posterolateral (PL) approach for 12 patients. The surgical procedures were performed by 3 senior surgeons—experienced in both the AL-S and PL approaches. All the acetabuli were reamed using same-size reaming, before cup insertion according to the manufacturer’s protocol. Adjunctive screw fixation was performed at the surgeon’s discretion if the initial fit was not satisfactory. The target cup position was at 40° abduction and 20° anteversion.

The cup diameters ranged from 44 to 60 mm (44 mm, 4 cases; 46 mm, 7 cases; 48 mm, 20 cases; 50 mm, 12 cases; 52 mm, 4 cases; 54 mm, 5 cases; 58 mm, 2 cases; 60 mm, 1 case). In all cases, a highly cross-linked polyethylene liner (X3; Stryker, Mahwah, NJ, USA) was placed and a ceramic femoral head (BIOLOX Delta ceramic; Stryker, Mahwah, NJ, USA) and cementless tapered wedge femoral stem was used. The head diameters ranged from 28 to 36 mm (28 mm, 7 cases; 32 mm, 43 cases; 36 mm, 5 cases). An Accolade II 127° neck angle (Stryker, Mahwah, NJ, USA) was used in 49 cases and an Accolade II 132° neck angle (Stryker, Mahwah, NJ, USA) was used in 6 cases.

### Radiographic assessments

**Fig. 1:**
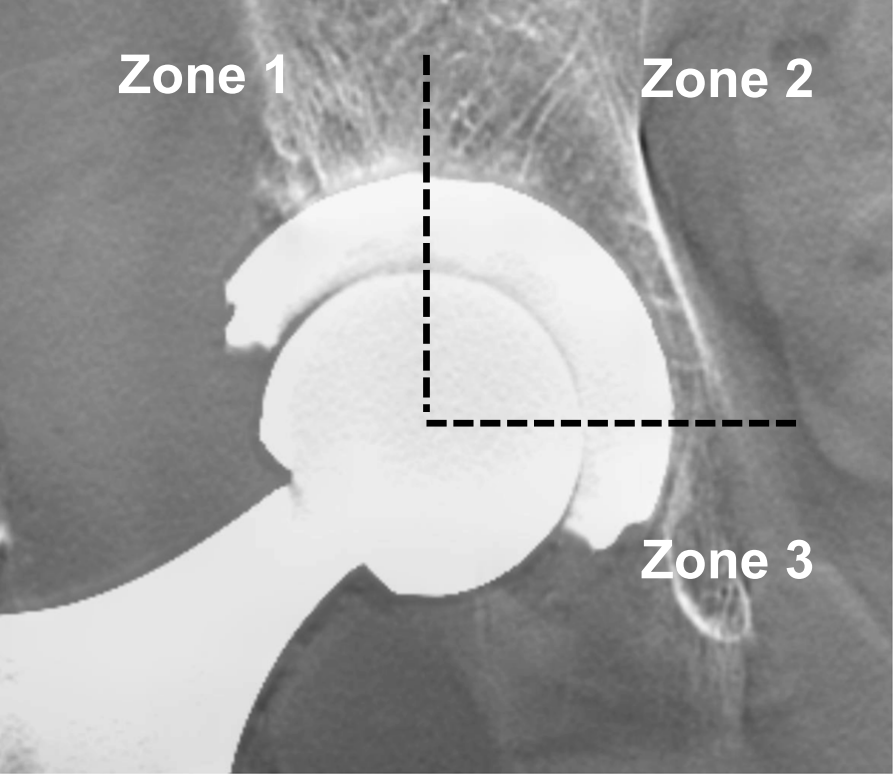
An example of a postoperative, pelvic, digital tomosynthesis anterior-posterior image divided into three zones by DeLee and Charnley

DTS (Sonialvision Safire 17, Shimazhu, Kyoto, Japan) and plain radiographs of the anterior-posterior (AP) view of the pelvis were taken preoperatively, and at 1 week, 3 months, 6 months, 1 year, and 2 years postoperatively. The center-edge (CE) angle and Sharp’s angle of the preoperative radiographs, and the cup abduction angle and the cup CE angle of the plain radiographs at 1 week postoperatively were measured. The RLs between the cup and acetabular rim on both DTS and plain radiographs at each postoperative checkup were measured according to the three zones defined by DeLee and Charnley [12]. The widths of the RLs were measured vertically, 45° diagonally, and horizontally from the center of the cup, in both the plain radiographs and the DTS. The measurements at each point were defined as zones 1, 2, and 3 for AP images (Fig. 1). The maximum width of the RL was measured in each case, and a RL ≥ 1 mm was considered significant as reported in the past [6]. All patients were classified into the RL (−) group or the RL (+) group. The RL (+) group was defined as those with RLs in any zone of the DTS at 2 years postoperatively, and the RL (−) group had no RL in any zone.

### Clinical assessments

Clinical evaluations based on the Japanese Orthopaedic Association hip disease evaluation questionnaire (JHEQ) were conducted preoperatively and at 2 years postoperatively, using self-administered questionnaires. The JHEQ is based on the lifestyle of people in Japan and has been widely used as a patient-reported outcome measure for hip joint diseases [13, 14]. The JHEQ questions evaluate pain (28 points), movement (28 points), and mental (28 points) subscales, with higher scores indicating a better outcome.

### Statistical analysis

Normal distributions of the demographic data, measurements of plain radiographs, and clinical scores were confirmed by the Shapiro-Wilk test. The comparison of age, BMI, CE angle, Sharp’s angle, and JHEQ between the RL (−) and the RL (+) groups was performed using the Mann-Whitney *U* test and paired *t*-test. The parameters of sex, diagnosis, and operative approach in the RL (−) and the RL (+) groups were analyzed using the chi-square test. The comparison of the number of cases with one or more RLs, between DTS and plain radiography, was performed by the chi-square test. Furthermore, logistic analyses were performed to evaluate the preoperative factors, the postoperative cup position, and a postoperative clinical evaluation regarding the factors associated with RLs at the 2-year point on the DTS. Data input and calculations were performed with SPSS version 22 (SPSS Inc., Chicago, IL, USA). In all analyses, *p* values < 0.05 were considered statistically significant.

## Results

The mean age of the patients at the time of surgery was 61.4 ± 13.4 years (range, 28–89 years), and the mean BMI was 24.6 ± 4.6 kg/m^2^. Forty-one patients had OA and 14 had ON. The average CE angle on preoperative radiography was 28.5° ± 18.0° and the average Sharp’s angle was 41.5° ± 5.7°.

**Fig. 2:**
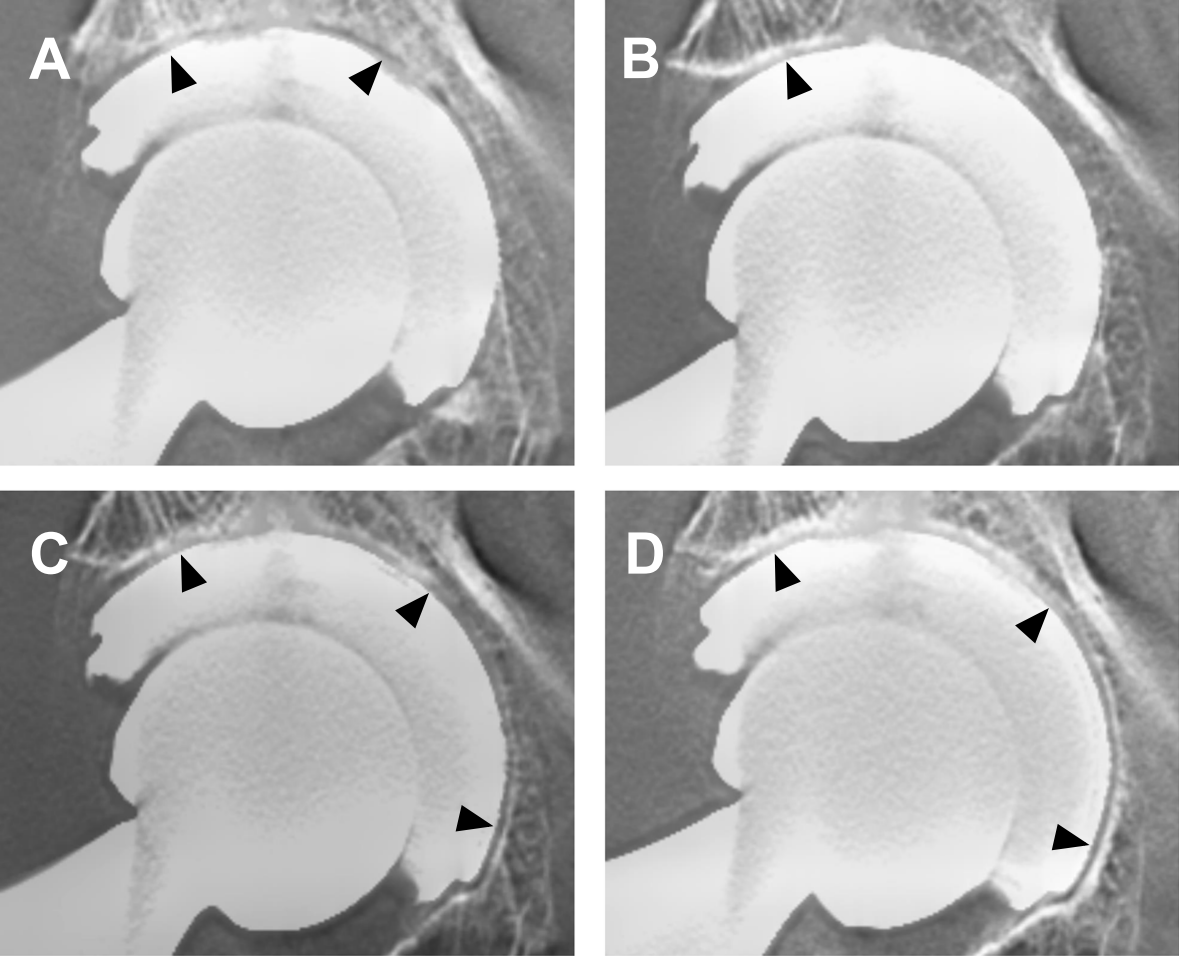
Digital tomosynthesis in an 83-year-old female after primary THA in the right hip. **a** At 1 week after surgery, there were RLs (black arrowheads) in zones 1 and 2. **b** At 6 months after surgery, there was an RL (black arrowhead) in zone 2. **c**, **d** At 1 year and 2 years after surgery, there were RLs (black arrowheads) in all 3 zones

**Fig. 3:**
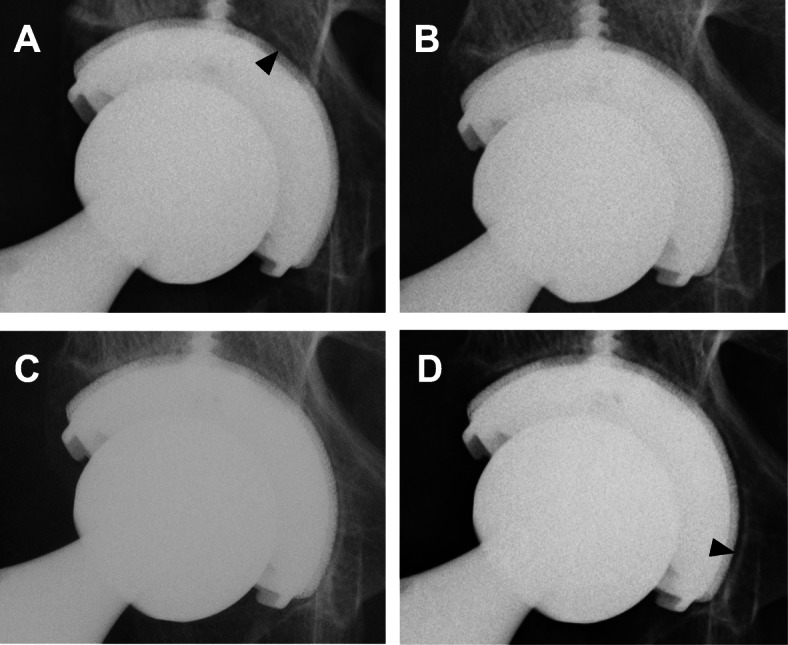
Plain radiographs of the same patient as in Fig. 2. **a** At 1 week after surgery, there was an RL (black arrowhead) in zone 2. **b** At 6 months after surgery, there was no RL. **c** At 12 months after surgery, there were no RLs ≥ 1 mm as considered significant. **d** At 2 years after surgery, there was an RL (black arrowhead) in zone 3

Radiographic evaluation during the 2-year follow-up period revealed that statistically, the number of cases with one or more RLs was greater on DTS than on plain radiography (Table 1). The number of patients with RLs in any one of the zones was 20 of 55 (45.4%) on DTS and 5 of 55 (9.1%) on plain radiography at 1 week postoperatively, and 20 (36.3%) and 5 (9.1%), respectively, at 2 years postoperatively. Statistically, there were more patients with RLs detected on DTS than on plain radiography at each postoperative point. At 2 years postoperatively on DTS, 11 of 55 cases (20.0%) had RLs in 1 zone, 5 cases (9.0%) exhibited RLs in 2 zones, and 3 cases (5.4%) had RLs in all 3 zones. On the plain radiographs, 4 cases of 55 (7.2%) had RLs in 1 zone, 1 case (1.8%) had RLs in 2 zones, and no case had RLs in all 3 zones. The DTS and plain radiographs for one case are shown in Figs. 2 and 3. On the DTS, there were at least one or more RLs from 1 week onwards up to 2 years postoperatively (Fig. 2). On the plain radiographs, there were RLs in zone 2 at 1 week postoperatively and in zone 3 at 2 years postoperatively (Fig. 3).

**Table 1:** Number of RLs ≥ 1 mm (%) on digital tomosynthesis and plain radiography by the zones of DeLee and Charnley at the designated postoperative periods

		1 week	3 months	6 months	1 year	2 years
Zone 1	DTS	15 (27.3)*	11 (20.0)	11 (20.0)	12 (21.8)	11 (20.0)
	RG	2 (3.6)	1 (1.8)	0	0	1 (1.8)
Zone 2	DTS	20 (36.4)	13 (23.6)	15 (27.3)	10 (18.2)	14 (25.5)
	RG	4 (7.3)	2 (3.6)	2 (3.6)	1 (1.8)	2 (3.6)
Zone 3	DTS	5 (9.1)	7 (12.7)*	9 (16.4)*	7 (12.7)*	5 (9.1)*
	RG	1 (1.8)	4 (7.3)	2 (3.6)	4 (7.3)	3 (5.5)
Total patients	DTS	25 (45.4)*	19 (34.5)*	17 (30.9)*	18 (32.7)*	20 (36.3)*
	RG	5 (9.1)	4 (7.3)	3 (5.5)	5 (9.1)	5 (9.1)

Percentages in brackets (%)

*RG* plain radiograph, *DTS* digital tomosynthesis, *total patients* number of patients with RLs in any one of the zone

*p* value less than 0.05 are shown as *p < 0.05 between RG and DTS

Depending on the results of the DTS at 2 years postoperatively, 35 cases were classified as the RL (−) group and 20 cases were classified as in the RL (+) group (Table 2). There were no statistically significant differences in the demographic data such as age (*p* = 0.554), sex (*p* = 0.161), BMI (*p* = 0.274), or diagnosis (*p* = 0.357), between the RL (−) and the RL (+) groups (Table 2). Radiologic evaluations showed a significantly smaller cup CE angle in the RL (+) group (19.6° ± 8.3°) compared with the RL (−) group (34.0° ± 8.5°) (*p* = 0.03). There were no differences in the preoperative CE angle, Sharp’s angle, and cup abduction angle between the two groups (Table 2). All domains of the JHEQ were also significantly improved in both groups (Table 3). There was no significant difference between the RL (−) and the RL (+) groups in the preoperative and the 2-year postoperative evaluation in each domain of the JHEQ. No revision surgeries were required.

**Table 2:** Comparison of demographic data and plain radiographic evaluations between the radiolucent lines (−) and the radiolucent lines (+) groups (mean ± standard deviation)

	RL (−) group (*n* = 35)	RL (+) group (*n* = 20)	*p* value
Age (years)	62.3 ± 12.0	60.0 ± 15.7	0.554
Sex male/female (*n*)	5/30	6/14	0.161
BMI (kg/m^2^)	24.7 ± 5.0	24.3 ± 4.0	0.274
Diagnosis OA/ON	25/10	16/4	0.357
Approach AL-S/PL	30/5	13/7	0.074
CE angle (degrees)	29.6 ± 19.1	26.5 ± 16.2	0.535
Sharp angle (degrees)	41.4 ± 5.7	41.6 ± 5.9	0.908
Cup diameter (mm)	49.0 ± 2.9	49.9 ± 4.1	0.786
Screw (*n*)	1.1 ± 0.8	1.5 ± 0.9	0.137
Cup abduction angle (degrees)	39.8 ± 4.5	40.1 ± 4.6	0.851
Cup CE angle (degrees)	29.7 ± 9.2	23.8 ± 10.4	0.030
Cup abduction angle 2 years postoperatively (degrees)	39.9 ± 4.5	40.1 ± 4.6	0.132

*RL* radiolucent line, *BMI* body mass index, *OA* osteoarthritis, *ON* osteonecrosis, *AL-S* anterolateral-supine approach, *PL* posterolateral approach, *CE* center edge, *RL* radiolucent line

**Table 3:** Comparison of clinical evaluations between the radiolucent lines (−) and the radiolucent lines (+) groups (mean ± standard deviation)

JHEQ	RL (−) group		RL (+) group	
	Pre	2-year	Pre	2-year
Pain	7.3 ± 6.1	22.5 ± 5.9*	8.4 ± 6.4	23.4 ± 6.4*
Movement	4.5 ± 4.4	16.5 ± 5.8*	7.0 ± 5.2	15.7 ± 7.0*
Mental	6.7 ± 6.8	18.6 ± 7.2*	10.5 ± 5.5†	18.9 ± 5.8*
Total	23.6 ± 11.0	58.0 ± 16.5*	25.9 ± 12.6†	58.0 ± 16.5*

*RL* radiolucent line, *Pre* preoperatively, *2-year* 2 years postoperatively, *JHEQ* Japanese Orthopaedic Association hip disease evaluation questionnaire

*p* value less than 0.05 are shown as *p < 0.05 between preoperatively and 2 years postoperatively and †*p* < 0.05 between the RL (−) group and the RL (+) group

The cup CE angle was significantly associated with the presence of RLs on DTS at 2 years after surgery, by the logistic analysis. The odds ratio for the cup CE angle was 0.92 (*p* = 0.021), suggesting that a decrease in the cup CE angle was associated with the presence of RLs. There were no significant associations between the presence of RLs at 2 years postoperatively on the DTS and age (*p* = 0.455), sex (*p* = 0.175), diagnosis (*p* = 0.301), BMI (*p* = 0.463), Sharp’s angle (*p* = 0.881), or the cup abduction angle (*p* = 0.907). Regarding the postoperative clinical evaluation, there were no significant associations between the presence or absence of RLs at 2 years on DTS and pain (*p* = 0.937), movement (*p* = 0.226), or mental status (*p* = 0.404) (Table 4).

**Table 4:** Factors related to the postoperative clinical evaluation for the presence of radiolucent lines ≥ 1 mm on digital tomosynthesis at 2 years postoperatively

	*B*	OR	CI	*p* value
Age (years)	−0.01	0.99	0.95–1.05	0.959
Sex male/female	−1.11	0.33	0.08–1.37	0.127
BMI (kg/m^2^)	0.07	1.07	0.94–1.22	0.327
JHEQ at 2 years after surgery				
Pain	0.01	1.01	0.88–1.14	0.937
Movement	−0.08	0.93	0.80–1.06	0.266
Mental	0.06	0.79	0.92–1.23	0.404

Statistical analysis—multiple linear regression analysis. Dependent variables—the presence of radiolucent lines ≥ 1 mm on digital tomosynthesis at 2 years postoperatively. Independent variables—age, sex, BMI, and JHEQ at 2 years after operations (pain, movement, and mental)

*B* nonstandardized correlation coefficient, *CI* 95% confidence interval, *BMI* body mass index, *JHEQ* Japanese Orthopaedic Association hip disease evaluation questionnaire, *OR* odds ratio

## Discussion

In the present study, at the 2-year follow-up, 20 of 55 (36.3%) cases had RLs of more than 1 mm in at least 1 of the zones defined by DeLee and Charnley. There was no difference in the patient-reported outcome scores between the groups with RLs less than 1 mm and RLs more than 1 mm at 2 years postoperatively. The cup CE angle was significantly associated with the presence of RLs at 2 years after surgery on DTS. In the follow-up up to 2 years postoperatively, more RLs were observed with DTS compared to plain radiography in all the zones.

In recent years, DTS has been introduced into clinical practice, and several studies reported on DTS assessments in the field of orthopedics [10, 11, 15–17]. This method can remove overlapping structures found on plain radiography and reduce metal artifacts found on CT, while providing in-depth information with high-quality images. Tang et al. reported that in their series of 48 consecutive patients scheduled for revision hip arthroplasty, the diagnostic accuracy for assessing the fixation stability of cementless hip arthroplasty by DTS was much higher than by plain radiography or CT [17]. Furthermore, they reported that the radiation dose of DTS examinations was slightly higher than with plain radiographs, but considerably lower than with CT. In this study, the findings of RLs between the cup and acetabulum could be detected by DTS from 1 week to 2 years after surgery. This suggests that DTS can better detect early gaps between implant and bone compared with plain radiography, and this method is an effective way to follow up THA patients in the postoperative period.

There were 2 previous studies that showed unacceptably high rates of RLs around Tritanium cups evaluated by plain radiography [6, 8]. Yoshioka et al. defined an RL as ≥ 0.5 mm and found RLs in 47 patients (36.1%) at 3 months after primary THA, increasing to 79 patients (60.7%) at the last follow-up (41.3 months). Carli et al. defined an RL as ≥ 1 mm and reported 109 cases of medium-term follow-up (4.24±1.49 years). They found RLs in 2 or more DeLee and Charnley zones in 30.3% and 3-zone involvement in 8.2% at 1 year postoperatively, and these proportions increased to 40.0% and 17.1%, respectively, at the minimum 5-year follow-up. In our study, on plain radiography at the 2-year follow-up, 4 cases out of 55 (7.2%) had RLs in 1 zone, 1 case (1.8%) had RLs in 2 zones, and no case had RLs in all 3 zones. There were fewer RLs than in previous reports, and the difference between this study and those two studies may have been influenced by the surgical technique. In previous reports, the cups were inserted with 1 mm under-reaming, and in the present study, they were inserted with same-size reaming. In previous studies, the Tritanium cup, which has a higher coefficient of friction than the conventional cup, was seated with under-reaming, possibly resulting in gaps in the immediate postoperative period of fixation by partial contact between the cup and the acetabulum.

It was unclear how the small cup CE angle was associated with the presence of RLs 2 years after surgery in the THA using the Tritanium cup. In the present study, the cup CE angle in the RL (+) group was 23.8 ± 10.4°, which was significantly lower than 29.7 ± 9.2° in the RL (−) group, and significantly associated with the presence of RLs at 2 years after surgery on DTS. Yoshioka et al. reported a larger percentage of RLs than the present study, reporting a larger cup CE angle of 46.9° using the Tritanium cup than in the present study [8]. In the previous study, the minimum host bone coverage on the cup required for stable fixation in total hip arthroplasty was studied by plain radiography and 3-dimensional images [18]. From the evaluation of 151 cases that were all stable cups with a mean follow-up period of 48 months, the cup had a minimum CE angle of 14.2° and a minimum three-dimensional bone coverage of 61.2%. In the present study, the cup might have been stable enough to contact the acetabulum anteriorly and posteriorly. However, only antero-posterior radiographic data were evaluated. In lateral DTS, it was difficult to get the corresponding antero-posterior DTS in every patient at every point in time. Further long-term follow-up and 3-dimensional analyses would be necessary.

There were several limitations in this study. Notably, the sample size was small and there were no cases that needed revision due to cup loosening. Secondly, the follow-up period was only 2 years; a longer follow-up period is needed for assessing the RLs and evaluating the clinical outcomes of the cup. Further studies in larger populations with long-term follow-up are required to validate the clinical significance of our findings. Thirdly, there was no evaluation of the implant surface. In a small cadaveric study, Faizan et al. found that the radiolucencies observed on the section plain radiograph of the Tritanium cup could not always be correlated with the metal to bone gap [19]. Artificial radiolucencies may have occurred as the interaction between the plain radiographs and the unique 3-D porous cup. The effect of that interaction on DTS has not been evaluated in this study and remains unknown. Fourthly, although CT with metal artifact reduction (CTMAR) has gained more attention recently, this technique was not used in our study. In addition, better assessment would be possible with postoperative CT scans. CTMAR would be a technique with higher diagnostic performance, higher sensitivity, and better interobserver agreement [20]; however, it would be considered unsuitable as a general postoperative screening method due to the risk of radiation exposure and cost-effectiveness. Despite these limitations, this study clearly showed that DTS can detect evidence of RLs around the highly porous cup better than plain radiography. Furthermore, to the best of our knowledge, this is the first study to evaluate RLs around a highly porous titanium cup (Tritanium) using DTS.

## Conclusions

In the short-term evaluation up to 2 years, statistically, there were more cases with one or more RLs around the highly porous titanium cups (Tritanium) detected on DTS than on plain radiography, at each postoperative point. The occurrence of RLs was not related to the postoperative clinical evaluation. Plain radiography may underestimate the RLs around highly porous titanium cups more than DTS.

## Data Availability

The datasets used and analyzed during the current study are available from the corresponding author on reasonable request.

## Abbreviations

AL-S: Anterolateral approach in the supine position
CE: Center edge
CTMAR: CT with metal artifact reduction
DTS: Digital tomosynthesis
JHEQ: Japanese Orthopaedic Association hip disease evaluation questionnaire
OA: Osteoarthritis
ON: Osteonecrosis
RL: Radiolucent line
PL: Posterolateral
THA: Total hip arthroplasty
